# Investigation of Physicochemical Properties and Surface Morphology of Hydrogel Materials Incorporating Rosehip Extract

**DOI:** 10.3390/ma16176037

**Published:** 2023-09-02

**Authors:** Magdalena Kędzierska, Katarzyna Sala, Magdalena Bańkosz, Dominika Wroniak, Paweł Gajda, Piotr Potemski, Bożena Tyliszczak

**Affiliations:** 1 Department of Chemotherapy, Medical University of Lodz, Copernicus Memorial Hospital of Lodz, 90-549 Lodz, Poland; magdalena.kedzierska@umed.lodz.pl (M.K.); piotr.potemski@umed.lodz.pl (P.P.); 2 Department of Materials Engineering, Faculty of Materials Engineering and Physics, Cracow University of Technology, 37 Jana Pawła II Av., 31-864 Krakow, Poland; katarzyna.sala@student.pk.edu.pl (K.S.); dominika.wroniak@student.pk.edu.pl (D.W.); 3 Department of Sustainable Energy Development, Faculty of Energy and Fuels, AGH University of Science and Technology, 30 Mickiewicza Av., 30-059 Krakow, Poland; pgajda@agh.edu.pl

**Keywords:** hydrogels, rosehip extract, roughness, sorption capacity

## Abstract

Hydrogel materials are used in many fields of science and industry. They are of particular importance in biomedical applications. In this work, hydrogels were obtained that could act as a dressing for wounds, at the same time being a carrier of substances with antioxidant activity. The discussed materials were obtained in the field of UV radiation. The correlation between the amount of photoinitiator used and the physicochemical properties and surface morphology of the obtained materials was investigated. In addition, the hydrogels have been incorporated with wild rose extract, which is characterized by antioxidant and anti-inflammatory effects. The analysis of the sorption capacity confirmed that the obtained material is able to absorb significant amounts of incubation fluids, which, in terms of application, will enable the absorption of exudate from the wound. The highest stability of materials was noted for hydrogels obtained with the use of intermediate amounts of photoinitiator, i.e., 50 µL and 70 µL. In the case of using 20 µL or 100 µL, the photopolymerization process did not proceed properly and the obtained material was characterized by a lack of homogeneity and high brittleness. With the increase in the amount of photoinitiator, an increase in the surface roughness of hydrogel materials was confirmed. In turn, spectroscopic analysis ruled out the degradation of materials in incubation fluids, indicating the potential for their use in biomedical applications.

## 1. Introduction

Hydrogels are materials with valuable properties that have found wide applications not only in medicine and pharmacy, but also in forestry and horticulture [1,2,3]. They consist of polymers, which are multi-molecular substances. These are polymeric nets that can store a significant amount of water or bodily fluids. The properties of hydrogels depend on many factors, including chemical composition, degree of cross-linking of the polymer, reaction temperature, ratio of reagents and additives, e.g., in the form of extracts [4,5]. Their most important properties include high sorption capacity, lack of toxicity and high flexibility. Due to their unique properties, hydrogels are the subject of many scientific studies [6,7]. M. Dziadek et al. focused on the use of 2,3,4-trihydroxybenzaldehyde (THBA) as a binding agent in CS-based hydrogels. Materials were evaluated for microstructure, mechanical properties, thermal behavior, swelling and degradation, in vitro mineralization process, antioxidant activity and cytotoxicity to normal and cancer cells. The THBA binding process significantly improved the mechanical properties of hydrogels, increased their ability to swell and slowed their degradation. In addition, THBA showed high antioxidant activity and selectively inhibited the growth of cancer cells without damaging normal cells. Additionally, the addition of rosemary acid increased the antioxidant and antitumor activity of hydrogel and promoted the mineralization process. This confirms that these hydrogels can be used in various areas of tissue engineering, including bone regeneration and post-tumor resection [8]. Next, in an effort to control the gel structure and viscoelastic properties, Belousov et al. conducted a study where they created 10 different variations of pectin-based hydrogels by adjusting the polymer concentration and the degree of esterification (DE) expressed as the number of free carboxyl groups. The study highlighted the ability to easily adjust the viscoelastic properties, porosity, water retention capacity and the ability to immobilize various substances of the hydrogels by changing the percentage of free carboxyl groups and pectin concentrations. This precise control over the hydrogel properties holds significant value in the treatment of glioblastoma [9]. On the other hand, after examining Ph-conjugated polymer hydrogels (hydrogels and their reaction with hydroxyl phenolic (Ph) groups), Ahmadian et al. noted that these hydrogels possess suitable mechanical integrity and the ability to control cell behavior. This study highlights that not a single trait, but a combination of mechanical characteristics, surface morphology, the presence of cell-binding sites and an extracellular matrix (ECM)-like network collectively influence fibroblast proliferation [10].

Various naturally derived biomaterials, including hyaluronic acid, collagen, alginate, chitosan and pectin, have been extensively investigated for forming hydrogels in tissue engineering [11,12]. Equally many studies have been carried out using various types of plant extracts as modifiers of hydrogels [13,14,15]. The chemical nature of hydrogels can vary depending on the specific components used in the polymerization process, allowing these materials to be adapted to different applications. In the present study, hydrogels were obtained via photopolymerization. Hydrogels obtained using photopolymerization have a chemical nature that results from the monomers, photoinitiator, crosslinking agent or other additives used. The polymer network of such systems forms a three-dimensional structure maintained by chemical bonds and physical interactions between the main polymer chains. In addition, these hydrogels are characterized by the possibility of designing their properties depending on the desired applications. This mainly concerns sorptive properties, hydrophilicity and mechanical properties. Such hydrogels are widely used in medicine, pharmacy and cosmetology, for example, as drug delivery systems, innovative dressing materials and tissue engineering scaffolds [16,17,18].

In this work, the use of an extract derived from wild rose fruit is proposed. Rosehip extract, derived from wild roses, is a valuable natural ingredient due to its richness in vitamins, minerals and antioxidants. It is particularly known for its high content of vitamin C, which helps to brighten the skin, neutralizes free radicals and contributes to improving skin tone [19,20,21]. Polyphenols and flavonoids found in plant extracts have been associated with health benefits, including protection against diseases such as cancer, diabetes and cardiovascular problems. The mechanisms of action of bioactive compounds from plant extracts on bacterial cells involve cell wall degradation, membrane destabilization, inactivation of intracellular enzymes and interference with nucleic acids. Cendrowski et al. studied the antioxidant and antimicrobial properties of freeze-dried extracts of rose hips (Rosa rugosa) obtained using various extraction techniques. Among the extracts, the aqueous extract of rose fruits showed the most significant inhibitory activity against the majority of the tested bacterial strains [22]. Next, the roses, rich in phytochemicals, were studied by Song et al. for their potential health benefits. Rose petal extracts (RPE) have shown anti-inflammatory effects [23]. Atopic dermatitis (AD), a skin disease caused by allergic immune reactions, adversely affects the well-being of patients, affecting their social and mental state. Choi et al. studied the effect of rose extracts on the care of atopic skin. Its healing effect has been confirmed [24]. Considering the importance of wild rose and hydrogel materials, an attempt was made to synthesize dressing materials modified with an antioxidant extract. The hydrogels were obtained in the photopolymerization process. The effect of the amount of photoinitiator on the physicochemical properties and morphology of the obtained materials was determined. A comparative analysis of materials containing wild rose extract and unmodified materials was also carried out. A detailed physicochemical analysis and imaging of the surface along with the determination of its roughness are carried out in this work. The novelty of this work concerns the combination of modern hydrogel materials obtained via an economical and ecological method of synthesis such as photopolymerization with rose extract, with excellent health-promoting properties. Such a combination makes it possible to obtain a material with increased durability and appropriate strength properties as well as showing anti-inflammatory and antioxidant effects. Hydrogel materials have been designed to obtain modern multifunctional dressing materials acting topically in the treatment of oozing and difficult-to-heal wounds.

## 2. Materials and Methods

### 2.1. Materials

Poly(vinyl alcohol) (PVA, crystalline powder, 87–89% hydrolyzed, Mw 13,000–23,000), polyvinylpyrrolidone (PVP, powder, average mol wt. 10,000), diacrylate poly(ethylene glycol) (crosslinking agent, PEGDA, average molecular weight Mn = 700 g/mol) and 2-hydroxy-2-methylpropiophenone (photoinitiator, 97%, d = 1.077 g/mL) were applied during the preparation of composite materials. All mentioned reagents were purchased from Sigma Aldrich (Saint Louis, MO, USA). Wild rose extract was purchased from eVitaldo company (Wrocław, Poland).

### 2.2. Synthesis of Hydrogel Materials

Synthesis of hydrogel materials was carried out by preparing solutions of PVP (polyvinyl pyrrolidone) and PVA (polyvinyl alcohol) in appropriate concentrations with a magnetic stirrer. A crosslinking agent (poly(ethyl glycol diacrylate)) and a photoinitiator (2-hydroxy-2-methylpropiophenone) were then added to reaction vessels. For modification, 3 mL of rosehip extract was added to the remaining mixtures. The thoroughly mixed mixture was then transferred to Petri dishes and subjected to UV field polymerization for 2 min for each hydrogel using a 180 W EMITA VP-60 lamp (Famed, Łódź, Poland) with a wavelength of λ = 320 nm. After completion of the polymerization process, the finished hydrogels were removed from the Petri dishes and discs of 1 cm diameter were cut and allowed to dry. The composition of each hydrogel is shown in Table 1.

**Figure 1:**
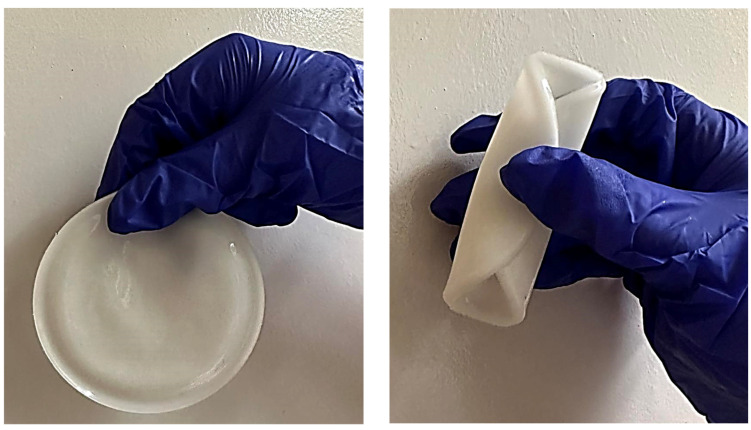
Pictures of rose-modified hydrogel materials (sample 50/3).

All matrices have been marked with the appropriate sample numbers and composition of the components, which will allow for further analysis and comparison. An example photo of a sample containing rosehip extract is presented in Figure 1.

### 2.3. Characterization of the Chemical Structure of Hydrogel Materials via Fourier Transform Infrared (FT-IR) Spectroscopy

Infrared spectroscopy was used to assess the absorption spectra using a Thermo Scientific Nicolet iS5 spectrometer equipped with an ATR attachment (Loughborough, UK). Changes in the composition of the material resulted from changes in the characteristic system of absorption bands. FT-IR spectra were recorded within the wavenumber range 4000–500 cm^−1^ (32 scans, resolution 4.0 cm^−1^). Both post-incubated and non-incubated samples were analyzed to assess possible degradation of the material. The analysis was performed at room temperature.

### 2.4. Sorption Capacity Analysis

The aim of the analysis was to thoroughly investigate the sorption capacity of hydrogel matrices in the context of swelling. The relevance of this study arises from the need to evaluate the potential use of hydrogel dressings for effective absorption of medicinal fluids. The analysis was based on the use of previously prepared and dried hydrogel discs. In the first step, the weight of each sample was accurately measured and then placed in a variety of incubation fluids, i.e., buffer phosphate (PBS), Ringer’s solution and distilled water. The next step was to incubate the sample in said liquids for a specified time, i.e., 1 h, 24 h and 72 h. After each period of time, excess liquid was drained from the disc surfaces and then the weight of the discs was accurately measured using a precise Radwag analytical balance (Radom, Poland). In order to minimize errors, each step of the analysis was carried out three times, which allowed for repeatable results. Finally, the swelling coefficient results obtained from the repeated tests were averaged to obtain a representative value of the sorption capacity of the hydrogel matrices. On the basis of the analysis, the degree of swelling was determined by determining the sorption coefficient α (Equation (1)).

(1) $$\alpha =\frac{m_{t}-m_{0}}{m_{0}}$$

where:

*α*—swelling ratio, g/g;
*m_t_*—mass of swollen sample after time “*t*”, g;
*m*_0_—mass of dry sample (before the study), g.

### 2.5. Incubation Studies in Simulated Body Fluids

The incubation test was performed to illustrate the interactions between the hydrogel matrix and solutions corresponding to human physiological fluids. Changes in pH values indicated the washout of non-crosslinked agents or caused, inter alia, degradation of samples in liquids. For testing, samples with a diameter of 1 cm were placed in sterile containers to which 50 mL of the above-mentioned liquids was added. The samples were incubated at 37 °C in an incubator. The pH values were measured using a CX-701 ELMETRON multifunctional (Elmetron, Zabrze, Poland) device every other day for 10 days, respectively.

### 2.6. Observation Using an Optical Microscope

To analyze the surface and structure of the synthesized hydrogel matrices, observations were made using Delta Optical Genetic Pro optical microscope with ×40 magnification (Delta Optical, Warsaw, Poland). The main objective of these observations was to visualize and compare surface changes resulting from modification of the amount of photoinitiator or introduction of additional components into hydrogel matrices.

### 2.7. Determination of the Roughness Profile Using a Digital Microscope

Next, an advanced VKX-7000 Keyence digital microscope with Keyence, capable of precise 4K imaging, was used (Keyence International, Mechelen, Belgium). This state-of-the-art microscope is equipped with CEO REMAX optical engine and 4K CMOS image sensor for high accuracy and magnification. The main objective of this step was to analyze the surface morphology of hydrogels and determine their roughness profiles using specific roughness parameters.

### 2.8. Analysis of Mechanical Properties

Strength analysis was performed to determine the mechanical properties of the hydrogels. Mechanical studies were performed in accordance with standards ISO 527-2 type 5A and ISO 37 type 2 [25,26]. As a result of the analysis, the tensile strength and percentage elongation of the tested materials were determined.

## 3. Results

### 3.1. Analysis of Sorption Capacity

**Figure 2:**
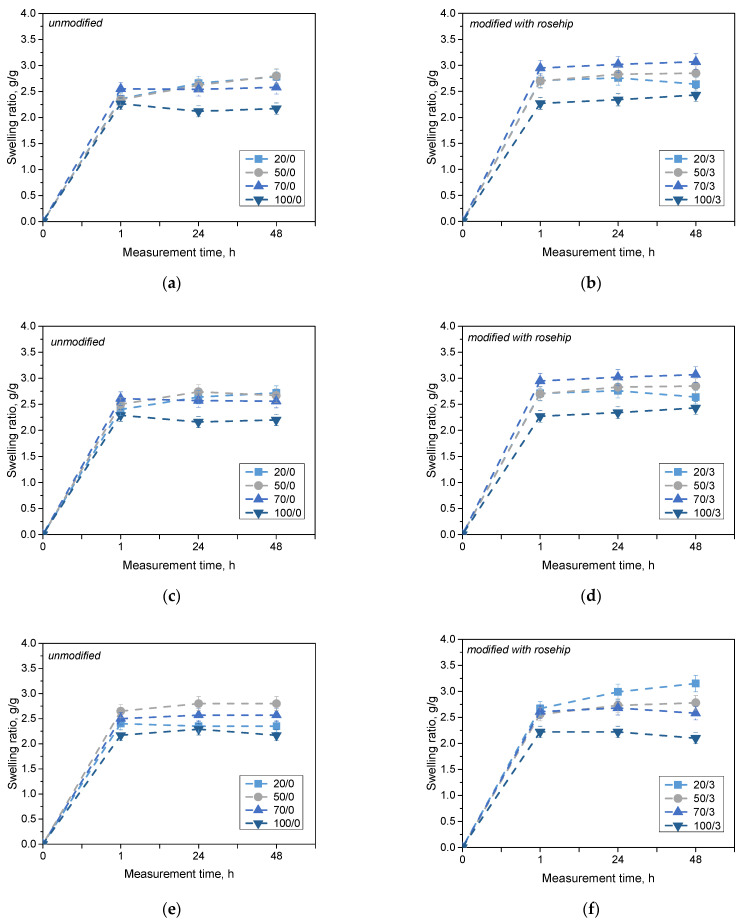
Results of sorption capacity analysis in distilled water (**a**,**b**), PBS liquid (**c**,**d**) and Ringer’s solution (**e**,**f**). The graph indicates the modified and non-extracted series. (*n*—number of repetitions, *n* = 3).

In order to investigate and compare the sorption capacity of hydrogel materials, swelling analysis was performed. The study considered the effect of the amount of photoinitiator added and the presence or absence of addition of rosehip extract. The results of the analysis for PBS liquid, Ringer’s solution and distilled water are presented in the following graphs (Figure 2).

The materials tested showed sorption capabilities in all tested samples and liquids. The greatest increase in the swelling rate was observed within the first hour of the study, after which the values stabilized and remained at a similar level. The swelling coefficients for all samples were in the range of 2 g/g to 3 g/g. The lowest values of swelling coefficients were observed using 100 μL of the photoinitiator. This effect was present in every fluid tested. This may suggest that the added amount of photoinitiator was too large, resulting in a brittle structure of the material, which was also observed during the synthesis of these materials. Too much photoinitiator adversely affected the strength of the samples, and their brittleness made it impossible to weigh the sample as a whole (the residue after disintegration of the disc was in liquid form). This resulted in a decrease in the weight of the test material and a decrease in the swelling coefficients for samples with 100 μL of photoinitiator added. For all materials with the addition of rosehip extract, higher swelling coefficient values were observed compared with unmodified samples. This phenomenon may result from the release of an additive in the form of a rosehip extract from a hydrogel matrix. When this substance is released from the polymeric material, the availability of penetrating fluid sites is increased, resulting in an increase in the swelling rate. In addition, rosehip extract consists of many components, including compounds with a strong hydrophilic character [27,28,29]. The presence of these hydrophilic groups may also contribute to an increased tendency to retain selected fluids through additional interactions such as hydrogen bonds [30]. The results of the statistical analysis for this study are presented in Table 2.

Statistical analysis clearly indicates the statistical significance when using a variable amount of photoinitiator. The photopolymerization process of polymer systems depends on many factors. However, one of the most important is the type and quantity of the initiator used. During synthesis, samples with the least amount of this agent decayed and indicated a disturbed proportion of the polymer matrix components. Incorrectly selected reagent ratios resulted in only partially polymerized materials which were easily degraded. Subsequently, samples containing the largest amount of photoinitiator interfered with the measurement. Difficulties in weighing them after decay had an impact on the reduction in swelling coefficients for samples containing 100 μL of photoinitiator. In this case, too much of this reagent resulted in too brittle a decomposable material. Other researchers have also observed significant relationships between the swelling coefficient and the amount and type of initiator used [31]. Importantly, it was also confirmed that, with the increase in sorption properties, the mechanical strength of materials obtained with different concentrations of photoactivator decreases [32,33]. The selection of suitable parameters thus makes it possible to obtain at the same time materials with adequate mechanical strength but characterized by a porous structure, allowing the absorption of liquids. The analysis confirmed that the best sorption properties with simultaneous preservation of the shape of the test samples were obtained for samples obtained from 50 and 70 μL of photoinitiator.

### 3.2. Results of the Incubation Study

**Figure 3:**
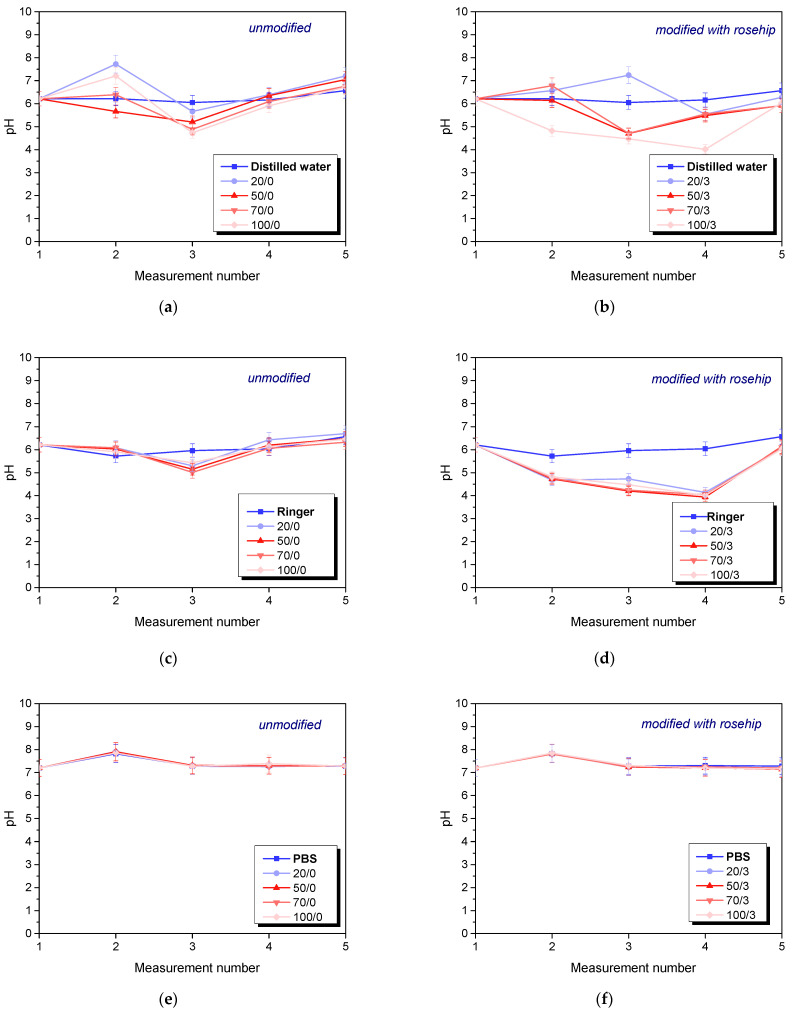
Results of incubation analysis in distilled water (**a**,**b**), PBS liquid (**c**,**d**) and Ringer’s solution (**e**,**f**). The graph indicates the modified and non-extracted series. (*n*—number of repetitions, *n* = 3).

An incubation test was performed to determine the hydrogen ion activity (pH) for the fluid in which the hydrogel matrices were incubated for 72 h. The results obtained are shown in the graphs below (Figure 3).

In the case of Ringer’s solution and distilled water, where significant pH changes were observed, this may indicate partial release or partial degradation of the test material components. These phenomena are more apparent with modified materials, suggesting a gradual leaching of acidic and alkaline plant extract components that affect pH changes. It should also be noted that the greatest pH changes were observed for samples containing the smallest (20 μL) and largest (100 μL) amounts of photoinitiator. These results are consistent with the results of the sorption capacities presented above. Again, the samples with the highest application potential contained an intermediate amount of photoinitiator.

### 3.3. Results of Antioxidant Activity Analysis

**Figure 4:**
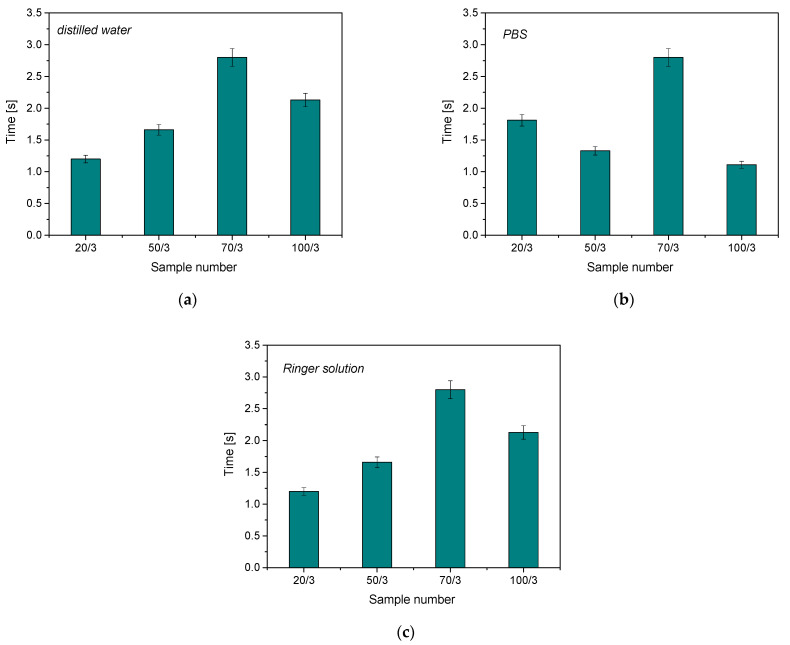
Results of antioxidant activity analysis of samples incubated in distilled water (**a**), PBS (**b**) and Ringer’s solution (**c**); (*n*—number of repetitions, *n* = 3).

The antioxidant activity study was conducted to determine the effect of hydrogels on simulated body fluids in the form of discoloration time in potassium permanganate solution. The results are below in Figure 4.

The compounds present in the plant extract reacted with potassium permanganate, causing a reduction in manganate ions and a change in the color of the solution. The greater the amount of Mn^VII+^ ion-reducing substances, the faster the color change and the higher the antioxidant properties of the test material. It was found that the addition of rosehip extract increases the antioxidant properties of the resulting hydrogel material, which is due to the broad chemical composition of the modifier used. The color change of the samples analyzed is presented in Table 3.

The color reaction indicates the reduction in manganate ions by the compounds present in the post-incubation fluid. For the results obtained, the color reaction was observed only for samples modified with wild rose plant extract. For unmodified samples, the color of the solution did not change over time. An incubation fluid containing no hydrogel material was used as an additional reference test. Again, no interaction between potassium permanganate and the solution was observed. It is also worth noting that the shortest decolorization time was observed for samples containing 20 μL and 100 μL of photoinitiator, respectively. As previously demonstrated, these samples were characterized by a partially degradable structure. Partial polymerization of the material and, thus, faster degradation of the material may have an effect on increasing the degree of release of the substances enclosed in the polymer matrix. Released compounds contributed more rapidly to the reduction in manganese ions. However, antioxidant activity has been proven for all materials obtained.

### 3.4. Optical Microscope Observations

Observation using an optical microscope was performed to visualize and compare the surfaces of the synthesized hydrogel materials. Images from microscopic observations are presented in Table 4.

Analyzing the presented photographs, it can be seen that both the addition of plant extract and the variable amount of photoinitiator affected the change in the surface structure of the studied material. Microscopic observations show that the unmodified wild rose extract series had a more wavy surface compared with the modified series. On the other hand, with the use of the plant extract, some surface smoothing was observed, which may be due to the deposit of the extract in the microscopic cavities of the polymer matrix.

In addition, for both the unmodified and wild rose extract series, the smallest surface undulation is noticeable using 20 μL of the photoinitiator, and the largest with 100 μL. This means that as the amount of photoinitiator increases, the surface corrugation also increases. It is also noteworthy that for a sample labeled as 100/3, which contained both the plant extract and the largest amount of photoinitiator, some irregularities and discontinuity of the matrix are observed. These observations are consistent with the results of the synthesis and sorption analysis, since it was this sample that showed the greatest brittleness and susceptibility to crumbling during the analyses carried out.

### 3.5. Roughness Profile Analysis

In order to evaluate roughness profiles and observe surface morphology, the tested materials were analyzed using a digital microscope. Images of the microscopic observations are presented in Table 5.

**Figure 5:**
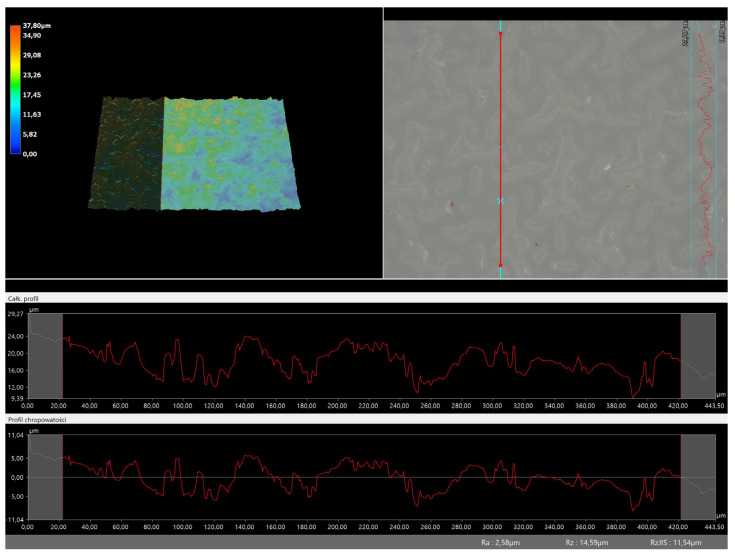
Roughness profile for hydrogel sample 20/0 (the red line marks the measurement point).

**Figure 6:**
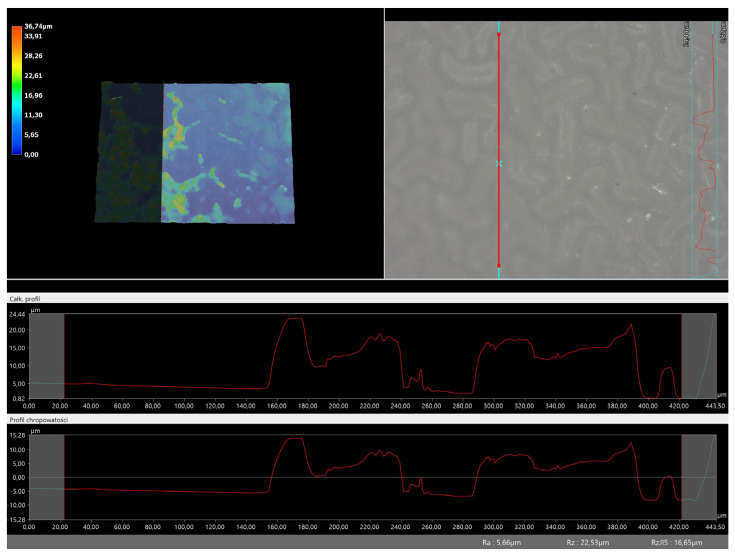
Roughness profile for hydrogel sample 50/0 (the red line marks the measurement point).

**Figure 7:**
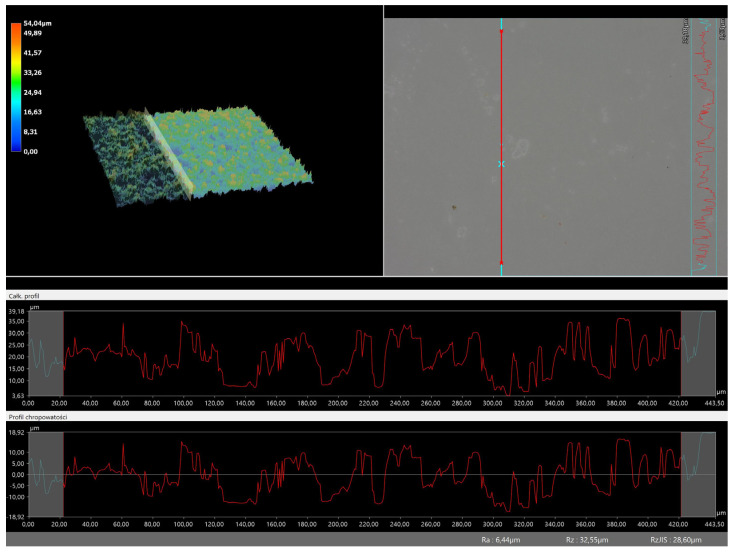
Roughness profile for hydrogel sample 70/0 (the red line marks the measurement point).

**Figure 8:**
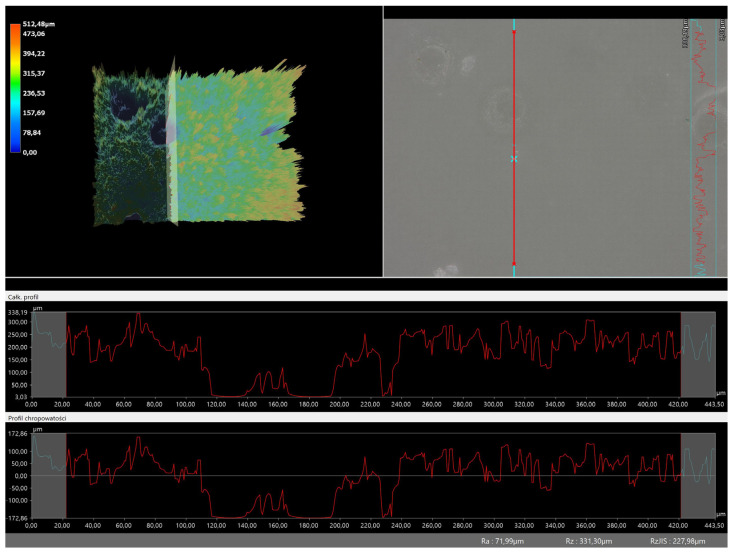
Roughness profile for hydrogel sample 100/0 (the red line marks the measurement point).

**Figure 9:**
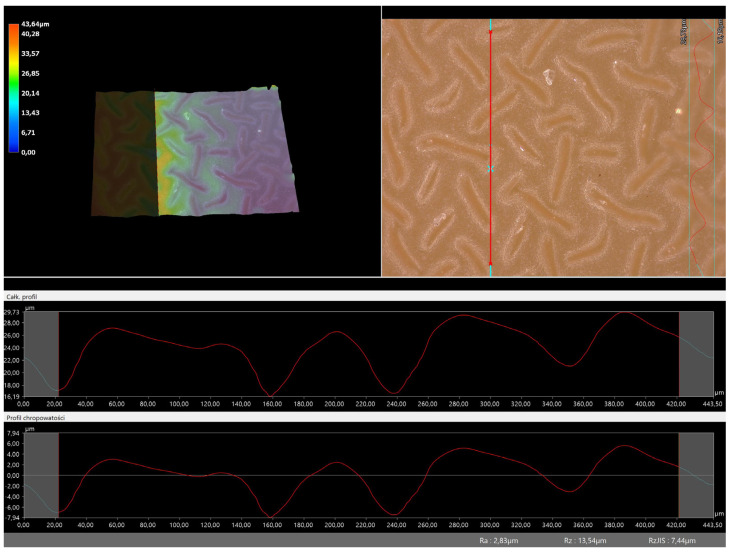
Roughness profile for hydrogel sample 20/3 (the red line marks the measurement point).

**Figure 10:**
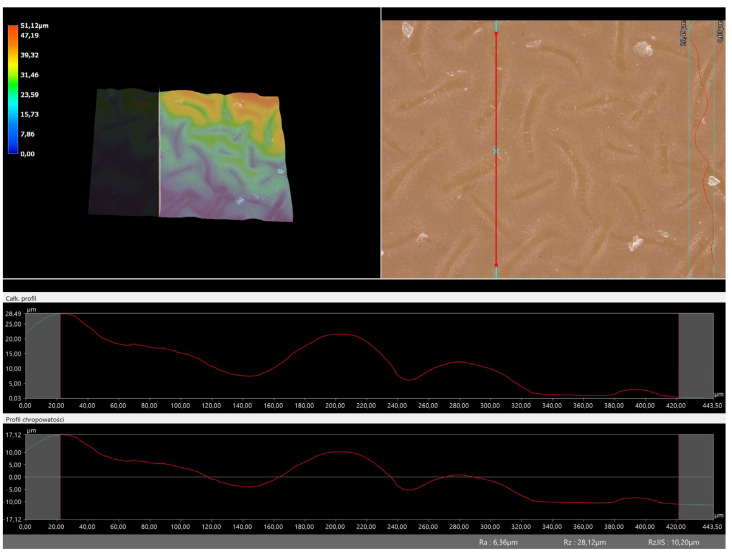
Roughness profile for hydrogel sample 50/3 (the red line marks the measurement point).

**Figure 11:**
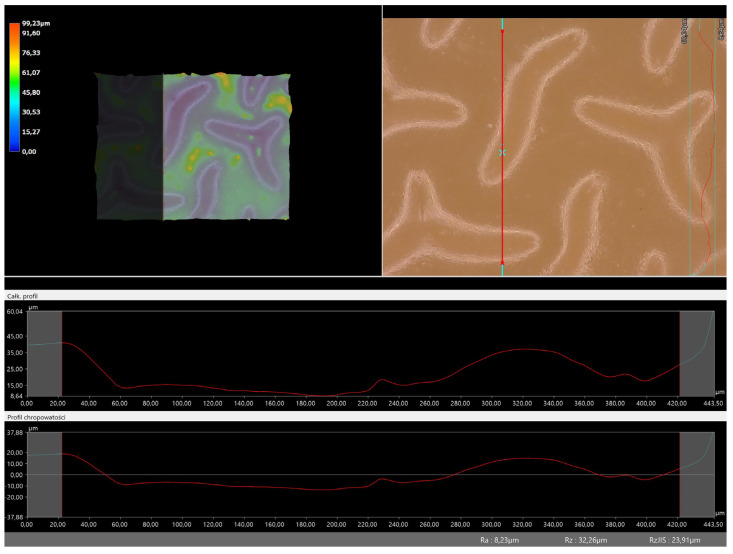
Roughness profile for hydrogel sample 70/3 (the red line marks the measurement point).

**Figure 12:**
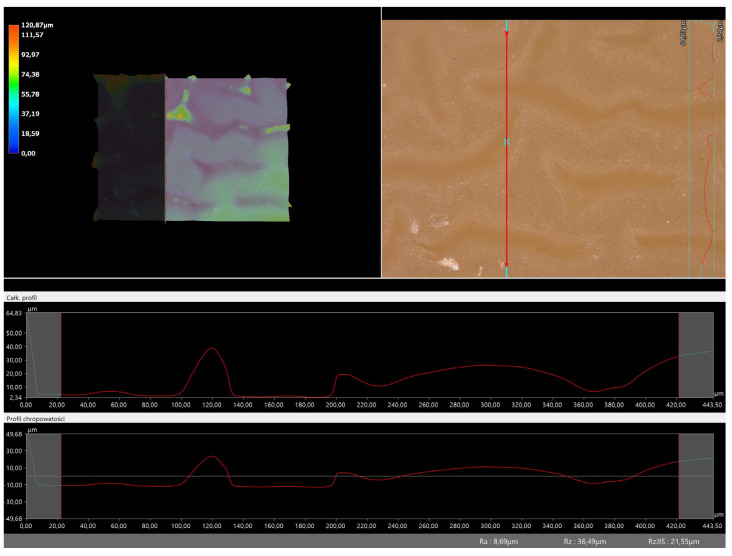
Roughness profile for hydrogel sample 100/3 (the red line marks the measurement point).

The photographs shown above are consistent with images obtained at lower magnification using an optical microscope. As the amount of photoinitiator increases, the corrugation of the hydrogel surface increases, which also increases its roughness. The roughness parameters along with the surface profile of the tested materials are presented below in Table 6 and Figure 5, Figure 6, Figure 7, Figure 8, Figure 9, Figure 10, Figure 11 and Figure 12.

Figure 5, Figure 6, Figure 7, Figure 8, Figure 9, Figure 10, Figure 11 and Figure 12 present the roughness profiles of the obtained hydrogel materials. The observed changes on the microphotographs were interpreted as indentations and undulations of the surface of the obtained materials. This was confirmed by an analysis of the roughness profile along with the color roughness scale used. These depressions are characteristic of the obtained hydro-gel materials, and their intensity depends on the composition of the synthesized materials. The roughness parameter Ra describes the arithmetic mean of the roughness in the profile. In the case of the tested materials, this parameter was in the range of about 2.5–9.0 µm. In the case of biomedical applications, materials must have appropriate properties. One of the key parameters affecting cell adhesion and proliferation is the surface and its properties. The surface roughness of an implant or other biomaterial has a significant impact on the adhesion of proteins and then cells to its surface. In the case of hydrogel materials intended for biomedical applications, this aspect is equally important. An environment conducive to cell profiling in the case of dressing materials promotes faster regeneration of damaged tissues. As demonstrated by Majhy et al., appropriate roughness affects cell growth. In the case of a rough surface, the cell membrane easily penetrates into the groove of the rough structure and easily anchors and then unfolds [34]. However, the best results are seen with intermediate roughness values. In the case of smooth surfaces, cell adhesion is limited, but also, surfaces with too much keratinization may prevent cell attachment [35]. Therefore, the obtained materials with a roughness parameter of about 8 μm have great potential as materials supporting cell adhesion and growth on their surface.

### 3.6. Results of Infrared Spectroscopy Analysis

**Figure 13:**
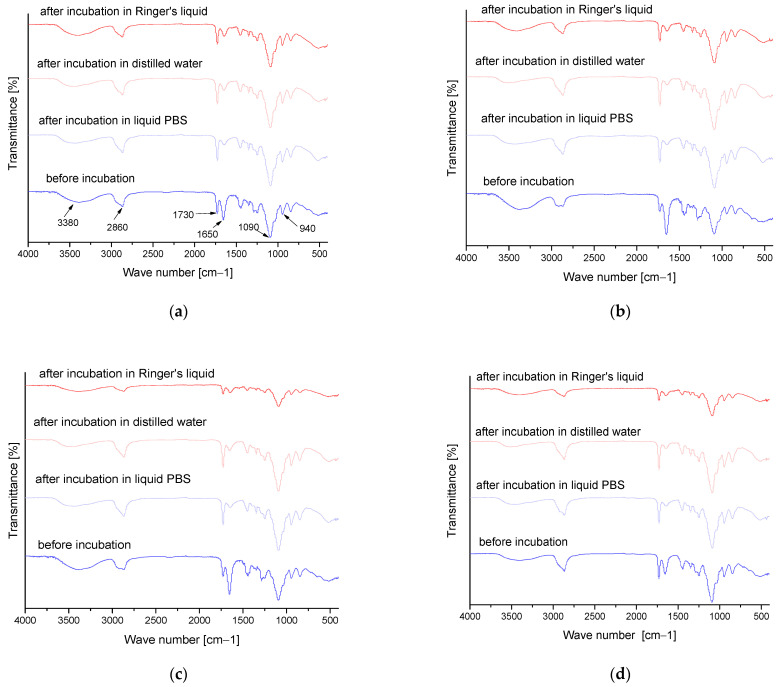
FT-IR analysis results for unmodified series (**a**) 20/0, (**b**) 50/0, (**c**) 70/0 and (**d**) 100/0.

**Figure 14:**
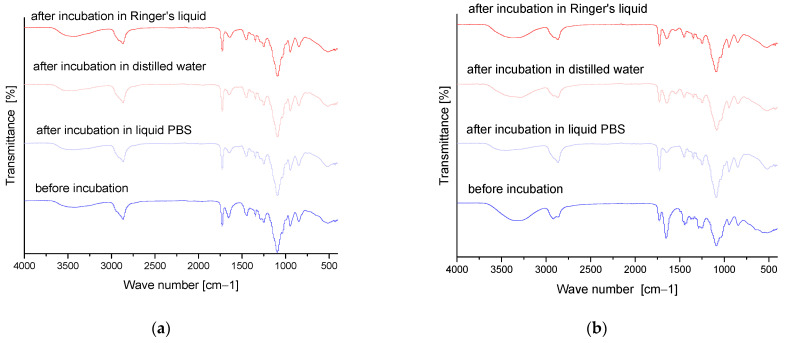

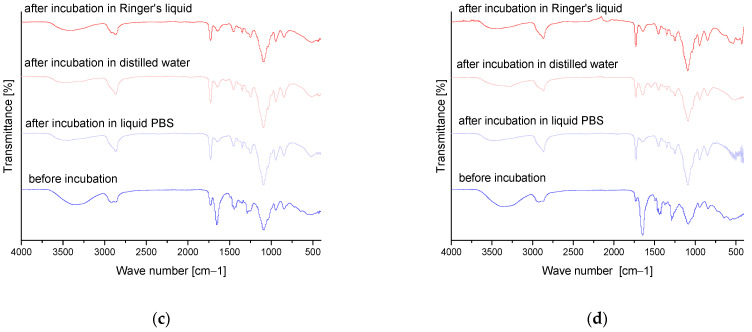
FT-IR analysis results for modified series (**a**) 20/3, (**b**) 50/3, (**c**) 70/3 and (**d**) 100/3.

FT-IR analysis was performed to determine the effect of the incubation process on the chemical structure of hydrogel matrices and to identify the functional groups present in them. The graphs (Figure 13 and Figure 14) show the results obtained.

Table 7 shows the identified absorption bands together with the corresponding type of vibration and characteristic bonds.

On the basis of the above graphs, the functional groups of the tested hydrogels and the effect of 4 days of incubation on their structure and possible degree of degradation were identified. The absorption bands shown in the FT-IR spectra plots were mainly derived from two substrates, i.e., polymer components such as PVP and PVA. The absorption band at a wavelength of 3380 cm^−1^ indicates the presence of an O-H tensile bond derived from poly(vinyl alcohol) [36,37,38]. In contrast, the peak band at 1650 cm^−1^ corresponds to the C=O tensile bonds present in the PVP structure [39,40]. In addition, bands derived from the crosslinking agent visible at a wavelength of 1730 cm^−1^ were also identified [41,42]. All presented FT-IR spectra are similar, and the absence of significant changes does not indicate degradation of the developed materials. The only significant difference is the decrease in the intensity of the absorption band attributed to the C=O bond, which occurs at a higher intensity for pre-incubation materials. This effect may be due to partial degradation or may also be related to the release of plant extract from the matrix during incubation. The C=O bond is characteristic of, inter alia, the polysaccharides and phenolic acids found in rosehip, and its partial loss may be associated with the washing of these substances from the polymeric material.

### 3.7. Results of Mechanical Property Analyses

**Figure 15:**
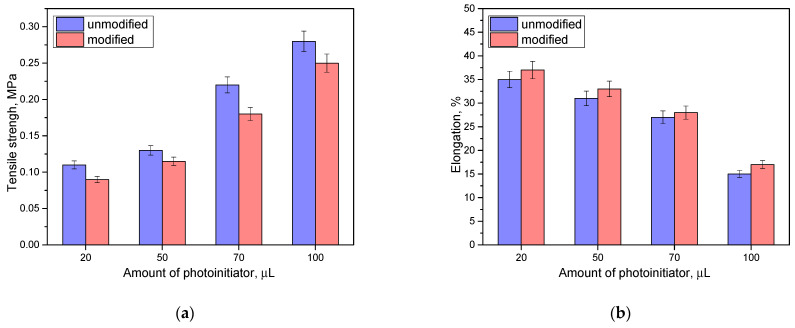
Results of mechanical property analysis: tensile strength (**a**); percent elongation (**b**). (*n*—number of repetitions, *n* = 3).

Next, the analysis of the mechanical properties was carried out. Figure 15 presents the results of tensile strength and percentage elongation of modified materials and those not containing rosehip extract.

Based on the analysis, it was found that the mechanical properties depended on both the amount of photoinitiator and the presence of rosehip extract. Tensile strength increases with the use of a higher amount of photoinitiator. With a higher concentration of this additive, there is an increase in the number of polymerization points, which can lead to a more compact structure with a higher density. Then, the strength of such material increases but, at the same time, its flexibility decreases. In the case of looser packing of the structure, which accompanies a smaller amount of photoinitiator, the elasticity of the materials is much higher (for the unmodified series, 20 µL of photoinitiator corresponds to an elasticity of about 34%, while for 100 µL in the same series it is twice as low). Then, it was noted that the presence of the modifying additive slightly deteriorates the strength properties while, at the same time, increasing the elasticity. The addition of rosehip extract, while keeping the values of the other components constant, can cause a slight dilution of the reaction mixture and loosening of the resulting polymer network, which indicates greater flexibility of such material.

## 4. Conclusions

- The chosen method of polymerization in the UV field allowed us to obtain hydrogel materials, which were modified by the addition of rosehip extract.
- All samples tested were sorptive and non-degradable. Samples modified with wild rose extract had the highest values of swelling coefficients, while hydrogel matrices containing 100 μL of photoinitiator had the lowest values.
- Incubation studies showed the greatest stability of the PBS buffer solution. Samples placed in distilled water and Ringer’s solution showed pH changes, suggesting the release of polymer matrix components.
- Hydrogel matrices enriched with rosehip extract were characterized by the rapid color change in the presence of KMnO_4_, indicating their antioxidant properties.
- Microscopic observations confirmed that increasing the amount of photoinitiator increases the surface corrugation of the hydrogel matrices, with the unmodified series showing greater corrugation compared with the rosehip extract series.
- Analysis of mechanical properties showed that with an increase in the amount of photoinitiator, the strength of these materials increases, while their elasticity decreases. The addition of rose extract, on the other hand, has the opposite effect, reducing the strength and increasing the percentage elongation of these materials.
- Analyses have confirmed that hydrogel materials based on PVA and PVP, and containing rosehip extract, show sorption, do not undergo rapid degradation in liquids simulating the environment of the human body and, additionally, have antioxidant properties. The described characteristics of the materials indicate their application potential in the field of biomedicine.

## Figures and Tables

**Table 1:** Composition of hydrogel materials.

15% PVP, [mL]	15% PVA, [mL]	PEGDA, [mL]	Photoinitiator [µL]	Rosehip Extract, [mL]	Sample Name
5	5	2	20	-	unmodified
					20/0
			50		50/0
			70		70/0
			100		100/0
			20	3	modified
					20/3
			50	3	50/3
			70	3	70/3
			100	3	100/3

**Table 2:** Statistical analysis of obtained data based on the two-way analysis of variance (ANOVA) with repetitions.

Independent Variable	Sum of Squares	Mean Square	f-Value	*p*-Value
Type of incubation fluid	0.71744	0.35872	1.46083	0.25195
Photoinitiator content	2.24704	0.74901	3.05022	0.04798
Interaction	1.70928	0.28488	1.16012	0.35968

At the 0.05 level, the population means of “type of incubation fluid” are not significantly different. At the 0.05 level, the population means of “composition of sample” are significantly different. At the 0.05 level, the interaction between both factors is not significant.

**Table 3:** Examples of color change of the tested samples.

Distilled Water (Without Sample)	Sample Unmodified Incubated in Distilled Water (50/0)	Sample Modified Incubated in Distilled Water (50/3)
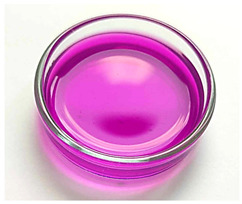	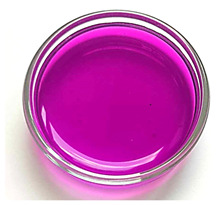	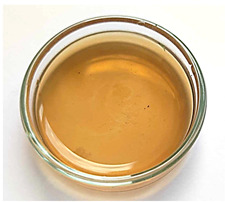

**Table 4:** Optical microscope images of unmodified and plant-extract-containing samples.

Photoinitiator [µL]	Unmodified Material	Modified Material
20	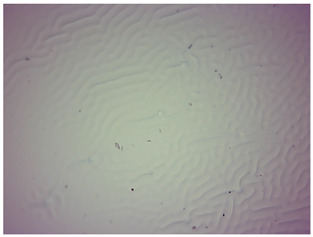	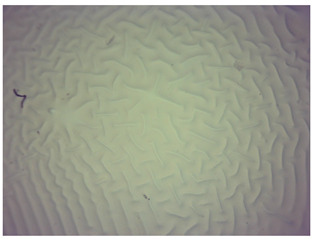
50	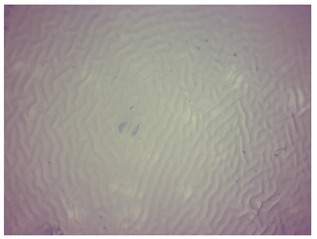	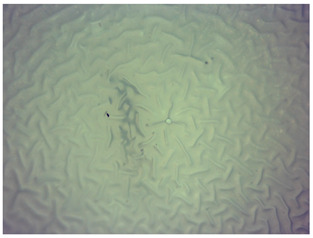
70	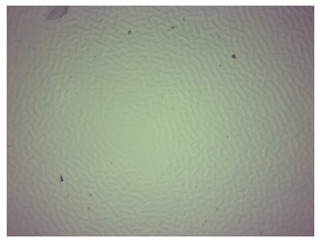	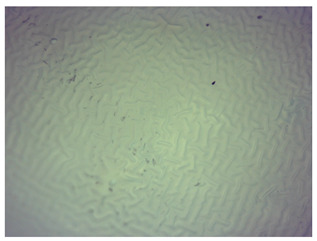
100	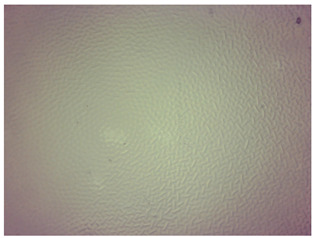	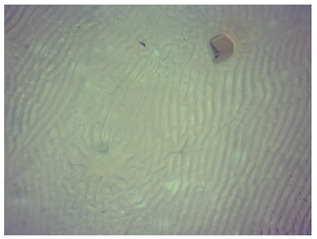

**Table 5:** Digital microscope images of unmodified and plant-extract-containing samples.

Photoinitiator [µL]	Unmodified Material	Modified Material
20	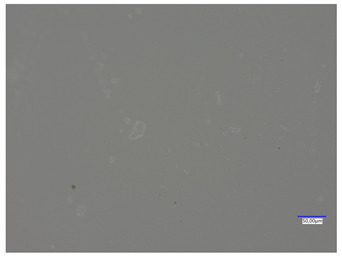	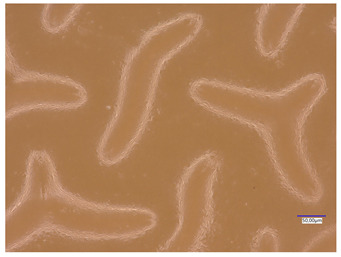
50	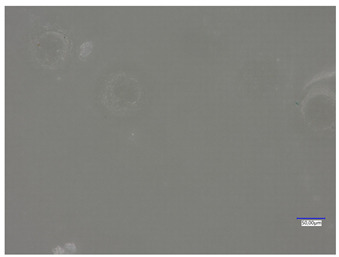	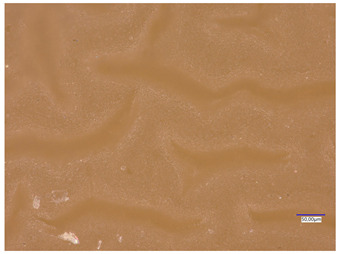
70	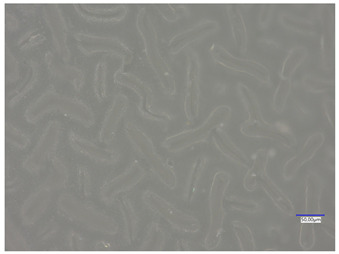	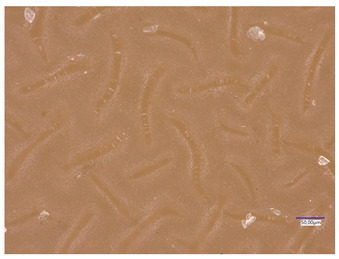
100	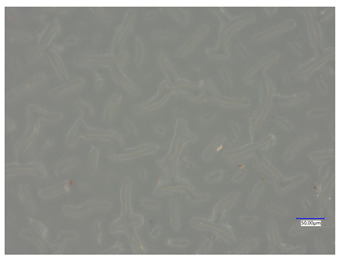	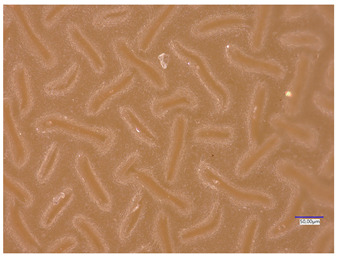

**Table 6:** Roughness parameters of hydrogel materials.

Photoinitiator [µL]	Roughness Parameter [um]	Unmodified Material	Modified Material
20	Ra	2.58	2.83
	Rz	14.59	13.54
	Rq	3.11	3.48
50	Ra	5.66	6.36
	Rz	22.53	28.12
	Rq	6.23	7.55
70	Ra	6.44	8.23
	Rz	32.55	32.26
	Rq	7.8	9.35
100	Ra	71.99	8.69
	Rz	331.3	36.49
	Rq	89.5	9.75

**Table 7:** Summary of characteristic bonds and vibrations for the obtained absorption band values.

Wave Number [cm^−1^]	Vibration Type	Characteristic Binding
3380 (3550–3200)	tensile	O-H
2860 (3000–2840)	tensile	CH2
1730 (1750–1735)	tensile	C=O
1650 (1705–1585)	tensile	C=O
1090 (1150–1085)	tensile	C-O-C

## Data Availability

Data sharing is not applicable for this article.
